# Are there optimal numbers of oocytes, spermatozoa and embryos in assisted reproduction?

**DOI:** 10.5935/1518-0557.20160032

**Published:** 2016

**Authors:** Tanya Milachich, Atanas Shterev

**Affiliations:** 1 SAGBAL Dr. Shterev, IVF Unit, Sofia, Bulgaria

**Keywords:** Oocyte number, Mature oocyte, Spermatozoa count, Assisted reproduction, Fertility preservation

## Abstract

The aim of this overview is to discuss the current information about the search for the optimum yield of gametes in assisted reproduction, as one of the major pillars of IVF success. The first topic is focused on the number of male gametes and the possible impact of some genetic traits on these parameters. The number of spermatozoa did not seem to be crucial when there is no severe male factor of infertility. Genetic testing prior to using those sperm cells is very important. Different methods were applied in order to elect the "best" spermatozoa according to specific indications. The next problem discussed is the importance of the number of oocytes collected. Several studies have agreed that "15 oocytes is the perfect number," as the number of mature oocytes is more important. However, if elective single embryo transfer is performed, the optimal number of oocytes will enable a proper embryo selection. The third problem discussed concerns fertility preservation. Many educational programs promote and encourage procreation at maternal ages between 20-35 years, since assisted reproduction is unable to fully overcome the effects of female aging and fertility loss after that age. It is also strongly recommended to ensure a reasonable number of cryopreserved mature oocytes, preferably in younger ages (<35), for which an average of two stimulation cycles are likely required. For embryo cryopreservation, the "freeze all" strategy suggests the vitrification of good embryos, therefore quality is prior to number and patient recruitment for this strategy should be performed cautiously.

## INTRODUCTION

This review paper discusses current literature publications regarding whether there is a specific mean number of oocytes, spermatozoa or embryos giving an optimal chance for pregnancy outcome after assisted reproduction, and whether the different methods chosen had benefits regarding better outcomes.

### 1. Male gametes

#### 1.1. Sperm number

With the development of intracytoplasmic sperm injection (ICSI) (Palermo *et al.*, 1992; Van Steirteghem *et al*., 1993a, b) and testicular sperm extraction (TESE) (Devroey et al., 1994; 1996; Tournaye *et al*., 1996), and with TESE-ICSI (Tournaye *et al*., 1997, Tesarik *et al*., 1994; Tesarik & Sousa 1995), the fathering possibility for patients with severe male factor infertility became a reality. Pregnancy could be even achieved by ICSI, even with totally immotile spermatozoa from the ejaculate or after electroejaculation in men with spinal -cord injury (Barros *et al*., 1997; 1998).

One of the first large series reporting ICSI outcomes came from Steirteghen (consisted of 1409 mature oocytes). They successfully fertilized 64.2% of the oocytes (Van Steirteghem *et al*., 1993b). A total of 67 pregnancies were achieved, of which 53 were clinical (pregnancy rate of 44.7% per started cycle and 49.6% per embryo transfer). The fertilization rate in this study was not influenced by the standard semen concentration characteristics. ICSI offers fertilization and pregnancy rates comparable to that achieved with normal sperm count (WHO, 1999) for couples who failed to achieve fertilization on repeated IVF cycles or had spermatogenesis disorders (surgically retrieved or by electroejaculation). Nevertheless, men with lower semen concentration [<5mln/ml] had an increased risk for aneuploidy in the resulting embryos (Campos-Galindo *et al*., 2015). Severe oligozoospermia was also associated with higher percentage of chromosomally abnormal spermatozoa (<1mln/ml: 31.4%) compared with good semen counts (>80mln/ml: 6.5%) (Rodrigo *et al*., 2014). Whether the morphology of human sperm is also important is the matter of the following discussion.

#### 1.2. Sperm selection

**Figure 1:**
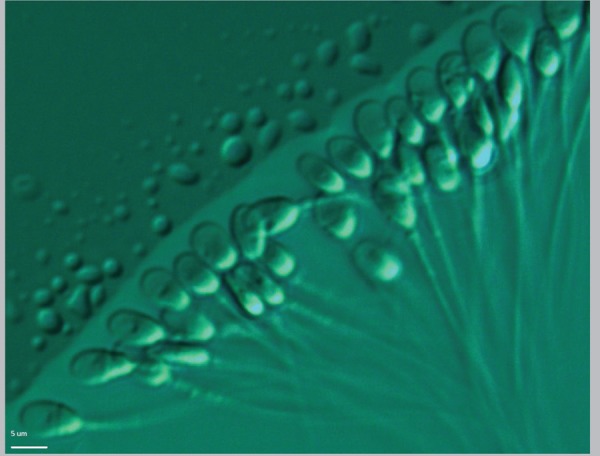
Selection after evaluation of motile sperm organelle morphology (MSOME) at x6000 magnification prior to ICSI.

Sperm morphology evaluation, as a routine diagnostic tool, seemed to be a powerful predictor of the fertilization potential of human spermatozoa under in-vitro and in-vivo conditions (Kruger *et al*., 1986; 1988), and most of the sperm defects were significantly more frequent in infertile than in fertile men (Sa *et al*., 2015; Auger *et al*., 2016). Twelve years after the introduction of intracytoplasmic morphologically selected sperm injection (IMSI) (Setti *et al*., 2013) procedure, based on the examination of motile sperm organelle morphology (MSOME), seemed to be effective in overcoming the late paternal effect and it is a promising real-time method for observation and selection of motile and morphologically normal spermatozoa (Figure 1) for intracytoplasmic injection (ICSI) (Vanderzwalmen *et al*., 2008; Cassuto *et al*., 2012).

Also the fertilization of oocytes and development of embryos with high implantation potential depends on the level of sperm DNA integrity (Franco *et al.*, 2012) and oocyte activation (Sousa & Tesarik, 1994). Several studies discovered that impairment of the morphological characteristics in sperm heads (big vacuoles above 50% of the head volume) had higher DNA fragmentation and aneuploidy rates compared with spermatozoa with normal heads (Pedrix et al., 2011). It was also suggested that sperm heads with big vacuoles could have detrimental effects in early embryo development (Vanderzvalmen *et al*., 2008; Cassuto *et al*., 2012) and negative association with fertility potential (Bartoov *et al*., 1994; Mundy *et al*., 1994). In order to decrease the miscarriage rate in couples with several attempts a study recommended the preselection of spermatozoa with lack of or with very small vacuoles (Berkovitz *et al*., 2006). Also the IMSI leaded not only to higher percentage but also to improved quality of resulting blastocysts on day 5 (Vanderzvalmen *et al*., 2008; Cassuto *et al*., 2012; Knez *et al.*, 2011), and had better implantation and pregnancy rates (Berkovitz *et al*., 2006). According to some authors, severe teratozoospermia was in correlation with lower fertilization and fragility of sperm DNA (Marci *et al*., 2013). The impact of IMSI on good embryo quality at day 3 and their availability for cryopreservation was also suggested recently (Dyulgerova-Nikolova *et al*., 2015). Despite those studies, some authors found that IMSI did not improve the outcome of patients with two successive IVF-ICSI failures (Gatimel *et al*., 2016), and more studies especially focused on different types of male infertility and the impact of IMSI procedure need to be performed. But as Vanderzwalmen & Fallet (2010) proposed: "Are there any indications to not select the best spermatozoa? Of course not."(Setti *et al*., 2013). Some other techniques, such as the selection of the fast motile spermatozoa during ICSI, may improve the qualities of the fertilizing spermatozoon by decreasing aneuploidy rates for chromosomes X, Y and 18 among preselected ones in men with severe teratozoospermia (Levron *et al*., 2013). Magnetic-Activated Cell Sorting (MACS) is also a promising method for obtaining sperm cells with non-apoptotic DNA material and, therefore, higher capability for fertilization and producing viable good quality embryos (Bucar *et al*., 2015). The method is based on the binding of superparamagnetic Annexin-microbeads to externalized phosphatidylserine (PS) at the outer leaflet of the sperm's plasma membrane with activated apoptosis signaling, or membrane damage (Grunewald & Paasch, 2013). The efficiency is still questionable and there is not enough information about the benefits of it in order to replace traditional sperm selection. Nevertheless, MACS could be useful in cases where poor sperm quality (oligo/astheno-zoospermia) and recurrent implantation failures were seen (Bochev *et al*., 2011) and thus, lead to improved clinical pregnancy rates (Bucar *et a*l., 2015).

Favoring selection of spermatozoa with intact DNA and normal nucleus, hyaluronan assay (HA) may be another alternative to optimize ICSI outcomes. In fact, some studies have shown significant differences (Parmegiani *et al*., 2010), while others did not find differences in clinical pregnancy rates or embryo quality (Majumdar & Majumdar, 2013). There is also a reduced pregnancy loss when combinations of both methods were applied (Paasch *et al*., 2007).

Birefringence still has some contribution in male infertility diagnosis and treatment. Some authors claimed that sperm head's birefringence could be used as a new criterion for sperm selection (Gianaroli *et al*., 2008). Although fertilization and cleavage rates did not differ between the study and control groups, in the most severe male factor condition, the rates of clinical pregnancy, ongoing pregnancy, and implantation were significantly higher.

#### 1.3. Impact of genetic changes in sperm

Currently, genetic testing is indicated primarily to elucidate the underlying diagnosis and to assess the risk to the offspring following successful treatment by e.g. ICSI, TESE etc. According to international guidelines, pre- and post-genetic test counselling by an appropriately trained professional is highly desirable, and in some countries even mandatory (Harper *et al*., 2014). There were also some major regulatory mechanisms that had gene defects associated (Pereira *et al.*, 2015), with specific morphological and motility abnormalities. An "indication threshold" for genetic testing is the total sperm count concentration below 10 million per milliliter (<10x106/ml). Recently, it has been estimated that about 25% of patients with azoospermia and severe oligozoospermia should undergo genetic testing. Molecular cytogenetics proved that lower sperm count was associated with higher level of autosomal and gonosomal aberrations (mainly represented in Klinefelter syndrome: 47, XXY, including various mosaics), that could be passed on to the next generation. Healthy children were born even from sperm from those men after adequate diagnosis and treatment (Madureira *et al*., 2014). Unfortunately, most of the cases have been first diagnosed with lower sperm count and male infertility and then the men underwent genetic testing.

The majority of men with chromosomal aberrations associated with infertility are apparently healthy males but with various forms of chromosomal translocations. They have higher risk of causing repeated miscarriages or stillbirth in their offspring, since their balanced translocations commonly get "unstable" by producing a variety of abnormal gametes (Alves *et al.*, 2002a; Harper *et al*., 2014).

Interestingly, some authors found that globozoospermia could be considered as "a new genomic disorder" (Elinati *et al*., 2012). The study confirmed that DPY19L2 was the major gene responsible for globozoospermia. To date, mutations in two genes, SPATA16 and DPY19L2, have been identified as responsible for this severe teratozoospermia.

In addition, approximately 1% of all infertile men were born with the congenital absence of vas deferens (CBAVD). Pathogenic mutations in the cystic fibrosis transmembrane receptor gene (CFTR) are associated with obstructive azoospermia due to CBAVD and, therefore, those patients are candidates for CFTR testing.

Various Y-chromosome deletions are predominantly found in non-obstructive azoospermia or severe oligozoospermia, and Sertoli cell only syndrome (Ferras *et al*., 2004; Fernandes *et al*., 2002; Kamp *et al*., 2001). Association of AZFa, AZFb, AZFbc and AZFc microdeletions with infertility is unambiguous. The majority of these deletions are in the AZFc region. Azoospermic men have a higher prevalence of microdeletions than oligozoospermic patients (Ferrás *et al*., 2004; Fernandes *et al*., 2006; Gonçalves *et al*., 2016). The data showed that no sperm could be retrieved in AZFa, AZFb, while there is a lower chance that viable sperm could be retrieved from AZFc cases.

In conclusion, genetic counselling should be offered to the family, as well as PGD or PGS, as a part of fertility treatment, since the underlying cause of male infertility is transferred to successive generations (Alves *et al*., 2002b; Pinho *et al*., 2005; Harper *et al*., 2014).

Future prospective studies for genetic treatment of male could be through flow cytometry cell sorting (FCCS) for separation of sperm cells (Vidal *et al*., 1998). This method is applied mainly for prevention of sex-linked genetic disorders (Dondorp *et al*., 2013). The method is based on staining of spermatozoa with specific markers and sorting them with flow cytometry, which provides a means of preventing significant disease in the offspring, and may help reduce the number of supernumerary affected embryos prior to preimplantation genetic diagnosis (De Geyter *et al*., 2013). 

### 2. Female gametes

#### 2.1. Oocyte number

Because of the limited number of oocytes available after ovarian stimulation, their count and quality seem to be of a great importance for advanced maternal age (AMA) and/or premature ovarian insufficiency (POI), with dramatically decrease of the number and implantation potential of oocytes together with a dramatic pick up of aneuploidy rates over 70% (NIHCE, 2013). IN addition, women with AMA have higher risk for miscarriages (over 40%) as well as drastically lower rates of clinical pregnancies.

If a natural cycle takes place in the beginning of IVF (Steptoe & Edwards, 1978), the likelihood of young women (<35) taking a baby home is only 3.8%; 1.3% (40-42 age) and 0% (>43 age) (HFEA, 2015). The live birth rates in natural cycle in poor responders and poor ovarian insufficiency (POR, POI) patients according to the Bologna criteria are significantly lower (2.6%) (Polyzos *et al.*, 2012). These findings can be explained by the "pregnancy loss iceberg" model, in which 60% of all obtained embryos are lost in pre/post implantation period and additionally, 15% of them drop out due to miscarriage, where over 40% are due to chromosomal aneuploidy (Azmanov *et al*., 2007). Some authors even called this iceberg as "the black box of early pregnancy loss" (Macklon *et al*., 2002).

Thus, in the last decade, different protocols for controlled ovarian hyperstimulation (COH) have been modified in order to optimize the number, quality and maturity of oocytes. Systematic reviews and meta-analyses have been published in order to evaluate stimulation duration, number of oocytes per egg retrieval and ongoing pregnancy rates (Bodri *et al*., 2011; Stimpfel *et al.*, 2015), and the risk of ovarian hyperstimulation syndrome (OHSS) (Sousa *et al*., 2015).

Several studies released by the European Society of Human Reproduction and Embryology (ESHRE) summarized that "15 oocytes is the perfect number" and suggested an optimal chance for achieving a pregnancy in one cycle (Sunkara *et al*., 2011), with the percentage going up to 37% when 15 oocytes had been collected. According to a large cohort study, the oocyte count is not as important as the optimal number of mature oocytes in metaphase 2 (mean number 9 and more metaphase 2) are collected (Bals-Pratsch *et al*., 2010). In cases of more than 16 oocytes retrieved, the pregnancy rate decreases slowly, but the risk for hyperstimulation increases drastically. Other authors found that the pregnancy rate per embryo transfer reached the absolute maximum (30.8%) when between 11 and 15 mature oocytes were available for ICSI injection (Steward *et al*., 2014) in a single embryo transfer (SET) strategy. Among the studies, there is another suggestion for so called "gold standard" with variation between 5-15 oocytes (Timeva *et al*., 2006). A different mean number of oocytes per collection have been proposed as optimal, but all studies agree that patient safety and health are the most important and the risk of OHSS should be evaluated carefully and minimized (Sousa *et al*., 2015).

#### 2.2. Oocyte morphology

Different types of dimorphisms in cumulus-oocyte complexes and in oocyte morphology (ooplasm, perivitelline space, first polar body and zona pellucida, giant oocytes) have been evaluated over the years (Mandelbaum, 2000), especially when the ICSI method was performed. This technique has enabled the precise assessment of oocyte morphology. There are many conflicting results in the literature concerning the incidence of oocyte dimorphism on fertilization rates, or potential further implantation and development. Most of the studies found an increased incidence of aneuploidy among dysmorphic oocytes (Van Blerkom & Henry, 1992; Kahraman *et al*., 2000).

**Figure 2:**
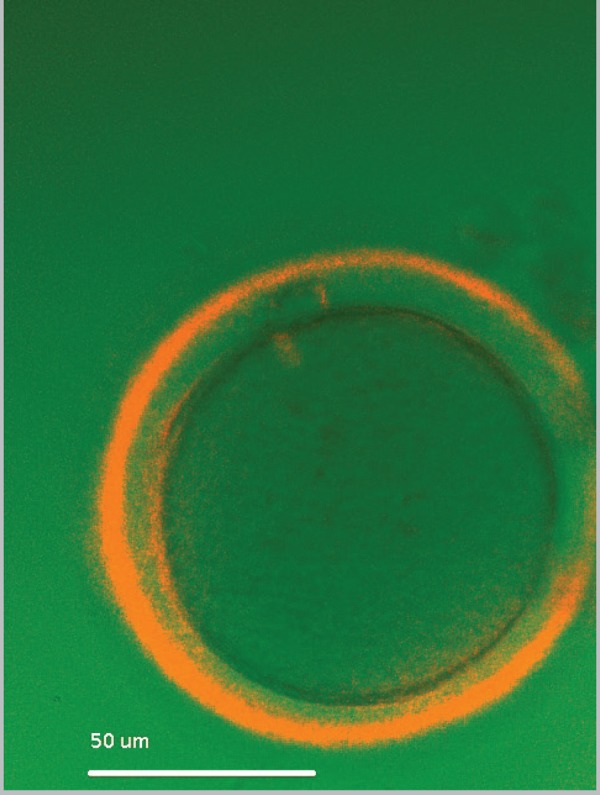
Evaluation of spindle position and zona pellucida birefringence of an oocyte at x300 magnification prior to ICSI.

Different equipment and software have been developed (birefringence/polarization microscopy) in order to evaluate the spindle position of mature oocytes (Figure 2). Some studies are focused on birefringent spindles and the prediction of fertilization rates after intracytoplasmic sperm injection (ICSI). The results indicated that the presence of a spindle in human oocytes can predict not only higher fertilization rates, but also higher embryo developmental competence (Wang *et al*., 2001). One study (Montag *et al*., 2011) criticizes the majority, claiming that they are only observational and not performed in a randomized manner, using other gamete selection markers for comparison. Despite that, polarization microscopy may help improve knowledge on meiosis. Whether or not certain applications such as spindle or zone imaging may lead to an increase in IVF success presently remains unclear.

### 3. Human preimplantation embryo genetics

The probability of obtaining optimal embryo quality and to have spare embryos for cryopreservation (CP) depends on oocyte and sperm count and quality. The chance for having a good blastocyst from one cohort of gametes increases if some other additional methods (e.g. MSOME) are applied.

Since its first application by Handyside *et al*. (1990) for an X-linked disorder in more than 1,000 healthy children born after preimplantation genetic diagnosis (PGD) and annual screening (PGS) (De Rycke *et al*., 2015). Sex-linked disorders, monogenic diseases, chromosomal abnormalities and translocations in either or both partners are some of the major indications.

The most challenging part of the PGD combined with HLA typing is the low probability of transfer in these cycles. Genetic counselors should provide the patient with the information that there is only 18.7% chance of finding an HLA-identical and healthy embryo, such as for beta-thalassemia (Milachich *et al*., 2013). Therefore, considerable numbers of oocytes and embryos are required in order to select disease-free and HLA-compatible embryos for transfer.

Blastocoel fluid and DNA extraction is the first non-biopsy method which was suggested for PGD or PGS purposes. Genomic DNA in human blastocoel fluid was already defined by the teams of Palini *et al.* (2013) and Gianaroli *et al.* (2014) although there are still some questions about representability of obtained results (Cohen *et al.*, 2013). Probably, the non-invasive PGS will spread in the future, together with non-invasive prenatal diagnosis and testing (NIPD, NIPT) (Milachich, 2014).

### 4. Human embryo morphokinetics

**Figure 3:**
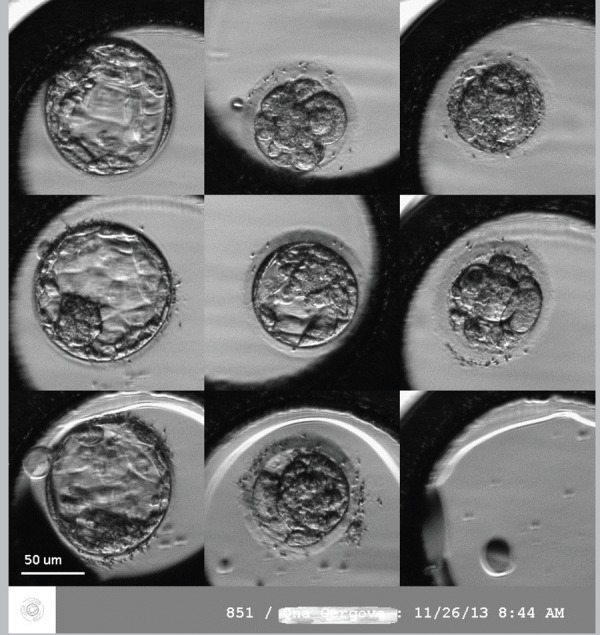
Time-lapse imaging of human embryos on day 5 (120 hours after in vitro fertilization).

The application of embryo time-lapse imaging (Figure 3) could be used as a predictor for good implantation and lower aneuploidy rate among transferable embryos. Much discussed studies (Meseguer *et al*., 2011; Campbell *et al*., 2013) reported that morphokinetics could be associated with the aneuploidy incidence. Embryo aneuploidy, a major cause of IVF failure, has been correlated with specific morphokinectic variables used previously to develop an aneuploidy risk classification model. The study by Campbell *et al.*, 2013, evaluated the effectiveness and potential impact of this model for unselected IVF patients without biopsy and preimplantation genetic screening (PGS), and discovered a significant difference in LBR between embryos classified with low, medium and high risk by demonstrating the clinical relevance of the novel aneuploidy risk classification model. There was a cautionary note against time-lapse imaging (Ottolini *et al*., 2014) and its inability to establish embryo aneuploidy risk.

A recent randomized controlled trial by Goodman *et al.* (2016) stated that the use of time-lapse morphokinectic data did not significantly improve clinical reproduction outcomes in all patients and in those with blastocyst transfers. Also, the findings from other systematic reviews did not support the routine use of time-lapse in clinical IVF. Therefore, future studies evaluating this technology in well-designed trials should be performed (Racowsky *et al*., 2015).

Although the use of time-lapse by itself is controversial and debatable, more and more studies are in favor of using this system for the selection of the most viable embryo to transfer, minimizing the early pregnancy loss from 25.8% to 16.6% (Campbell *et al.*, 2013).

### 5. Cryopreservation - count of oocytes and embryos

Major advances achieved in the past few years in the cryo-laboratory (e.g. vitrification) have brought about significant changes to the practice. Oocyte fertility preservation (OFP) for social or oncologic reasons, the possibility to create oocyte banks for egg donation programs, the opportunity to avoid ovarian hyperstimulation syndrome ("freeze all" embryos) or a strategy to accumulate oocytes in low-yield patients and then transfer embryos in a natural cycle are some of the options that are now available with the development of cryopreservation.

Each year of maternal age increase, decreases the delivery rate by 7%. Survival rate and female age reflect oocyte quality, which declines with age. Women at age 40 face a 40% chance of miscarriage if they can get pregnant at all, and by the age of 45, the risk of miscarriage is 75%. Freezing a woman's eggs around the age of 30 "freezes the time" her fertility potential, and gives her the chance of a healthy pregnancy at a time of her choosing (Lockwood *et al.*, 2011), but the role of oocyte cryopreservation in a real chance for obtaining pregnancy is still debatable.

Vitrification of oocytes from fertile young patients can produce high pregnancy rates. The inclusion criteria for OFP are: age (<35) at the time of recruitment; prior tubal ligation after the last child; body mass index <30 and basal antral follicle count >10. One of the recently proposed strategies (Devine *et al*., 2015) was to have patients undergo oocyte cryopreservation before the age of 35 years and to obtain at least 16 MII oocytes for potential use after the age of 40 years. Within this strategy, women attempt spontaneous conception by timed intercourse for a period of 6 months when reaching age of 40 years. If no spontaneous pregnancy is obtained, the women are then submitted to two IVF cycles using previously banked oocytes. Another multicentric prospective cohort study reported no differences concerning survival, fertilization, and embryo development, but with three determinants of success: patient age (<38 years), number of vitrified MII oocytes (≥8), and blastocyst stage on embryo transfer. Additionally, when the number of vitrified oocytes was higher, delivery rates increased from 22.6% to 46.4% (Rienzi *et al*., 2012). There is another study with comparable results where the mean number of vitrified oocytes was 7.2 (Cobo *et al*., 2012a). Other studies found that pregnancy rates per thawed oocyte varied between 4,5% and 15%, where most women were younger than 35 years. The overall pregnancy rate ranged between 36-61% (Practice Committees of American Society for Reproductive Medicine & Society for Assisted Reproductive Technology, 2013), but again the female age was strongly related with a higher chance for extending reproductive options (Cobo & Garcia-Velasco, 2016). A recent study found that the oocyte vitrification for elective fertility preservation was an efficient option when at least 8 to 10 metaphase II oocytes were collected and, thus a reasonable success was achieved (Cobo *et al*., 2016). Also, the live birth rate was correlated with the additive role of every collected oocyte (gain of 8.4% per additional oocyte) in the group of women below 35 years of age.

Unfortunately, very few studies reported clinical outcomes with vitrified oocytes in cancer patients. Progress in cancer treatment using radiotherapy or chemotherapy has improved survival rates among malignant diseases. This is particularly evident in children and breast cancer patients. For most oncologic patients there is a chronic adverse effect of radiation or cytotoxic chemotherapy, including gonadal failure and infertility, which often cause distress, low self-esteem and undermined quality of life. Thus, the need is evident for an effective OFP strategy that provides the chance to conceive a child with one's own gametes. Special care must be paid to any condition and to the decision on the number of oocytes to be stored. Patients must be counseled objectively, according to their possibilities and current evidence to avoid false hopes, especially in cancer patients, where interdisciplinary collaboration with oncologists, psychologists and gynecologists is required (Waldby, 2015).

Since AR is unable to fully overcome the effects of age on fertility loss after the age of 35 years and additionally, a higher proportion of maternal and/or fetal morbidity and mortality are associated with advanced maternal age. Therefore, many medical organizations promote educational programs that encourage procreation or egg freezing at a maternal age of 20-35 years. It was strongly suggested to advise and to inform patients that they should ensure a reasonable number of cryopreserved oocytes, for which more than one stimulation cycle is likely required. To date, there is good evidence to disregard oocyte vitrification and experimental warming since it had similar pregnancy rates as the use of fresh oocytes for IVF/ICSI procedure in the group of young patients (Practice Committees of American Society for Reproductive Medicine & Society for Assisted Reproductive Technology, 2013) for fertility preservation purposes.

Another recent strategy for improving IVF outcomes is the "freeze-all" embryo policy. Even in a group of patients that was selected for fresh ET (*P*≤1.5 ng/mL), implantation may be impaired by COH, and outcomes may be improved. COH may contribute to endometrium modifications, which might be related to poorer outcomes when fresh ET is performed. In cycles with fresh ET, there is still a risk of OHSS. Thus, this strategy was implemented for special cases: in which *P* was >1.5 ng/mL on the trigger day; women aged 20-45 years; fresh and frozen-thawed ET performed with good-quality embryos only (Roque, 2015). Some exclusion criteria were: patients with a history of recurrent pregnancy loss; implantation failure (≥3 previous attempts); antral follicle count ≤5; severe male factor infertility (oligospermia <1 million/mL, and azoospermia) (WHO, 1999); uterine pathology. Clinical pregnancy rate was lower (35,9%) in the fresh cycle group, whereas when the freeze all strategy was applied this rate went up (46.4%).

Another study (Cobo *et al*., 2012b) included a large cohort of patients (3,150 warming cycles), where the policy for embryo cryopreservation depended on the morphologic quality and only optimum and good quality embryos were cryopreserved in women with risk of OHSS, impaired endometrium pattern, or high progesterone levels. Thus the clinical pregnancy rate of vitrified day 5 embryo varies between 41.7% and 49.3% per transfer, respectively, according to their "optimal" or "very good" quality.

Further randomized clinical trials are needed to confirm the advantage of this strategy and determine for which group of patients the "freeze all strategy" would be most beneficial.

## CONCLUSION

In conclusion, the mature oocyte count, with maternal age and the proper sperm selection might be the major or dominate circumstances for obtaining better outcome in IVF/ICSI cycles, SET cycles, embryo cryopreservation and oocyte fertility preservation, but still further trials are needed in order to evaluate the role of each one of these factors.
